# Complexity Analysis of EEG Data in Persons With Depression Subjected to Transcranial Magnetic Stimulation

**DOI:** 10.3389/fphys.2018.01385

**Published:** 2018-09-28

**Authors:** Karolina Lebiecka, Urszula Zuchowicz, Agata Wozniak-Kwasniewska, David Szekely, Elzbieta Olejarczyk, Olivier David

**Affiliations:** ^1^Department of Automatics and Biomedical Engineering, AGH University of Science and Technology, Kraków, Poland; ^2^Nalecz Institute of Biocybernetics and Biomedical Engineering, Polish Academy of Sciences, Warsaw, Poland; ^3^U1216, Inserm, Grenoble, France; ^4^Grenoble Institut des Neurosciences, Université Grenoble Alpes, Grenoble, France; ^5^Service de Psychiatrie, Centre Hospitalier Universitaire Grenoble Alpes, Grenoble, France

**Keywords:** EEG, repetitive transcranial magnetic stimulation, complexity, Higuchi fractal dimension, major depression disorder, bipolar disorder, depression

## Abstract

**Aim:** The aim of this work was to study the neurophysiological effect of repetitive transcranial magnetic stimulation (rTMS) applied to the left dorsolateral prefrontal cortex (DLPFC) in 8 patients with major depression disorder (MDD) and 10 patients with bipolar disorder (BP), considering separately responders and non-responders to rTMS therapy in each of both groups.

**Methods:** The Higuchi’s Fractal Dimension (FD) was analyzed from 64-channels EEG signals in five physiological frequency bands and every channel separately. Changes of FD were analyzed before and after 1st, 10th, and 20th session of rTMS.

**Results:** Some differences in response to the rTMS therapy was found across individual groups. In MDD responders, FD decreased in all bands after longer stimulation (20th session). Whereas, in BP non-responders, FD decreased after 1st session in all bands as well as after 10th session in lower frequencies (delta and theta). In MDD non-responders and BP responders FD increased at the beginning of the therapy (1st and 10th session, respectively), but the final FD value did not changed in comparison to the initial FD value, except the FD decrease for theta band in BP responders. Comparison between groups showed a higher FD in MDD responders than in MDD non-responders in every band before as well as after stimulation. In contrast to MDD patients, FD was lower in BP responders than in BP non-responders in higher frequency bands (alpha, beta, and gamma) in both conditions as well as in lower frequency bands (delta and theta) after stimulation. Comparing both groups of responders, FD was lower in MDD than in BP in every band, except alpha. In case of non-responders, FD was higher in BP than in MDD in all bands in both conditions.

**Conclusion:** The results showed that FD may be useful marker for evaluation of the rTMS effectiveness and the therapy progress as well as for group differentiation between MDD and BP or between responders and non-responders. The changes of FD under the influence of rTMS allow to unambiguously conclude whether the effect of stimulation is positive or negative as well as allow to evaluate an optimal time of rTMS.

## Introduction

Major depressive disorder (MDD) is a mood disorder that causes a persistent feeling of sadness and loss of interest (Sadock et al., 2003; Hersen and Rosqvist, 2008; American Psychiatric Association, 2013; WHO, 2017). While, bipolar disorder (BD), called manic depression, is a mental health condition that causes extreme mood swings that include periods of abnormally elevated mood (mania or hypomania) and lows (depression) (Anderson et al., 2012; American Psychiatric Association, 2013; WHO, 2017). The causes of depression are divided into two main types: (1) endogenous – resulting from abnormal functioning of the central nervous system (CNS) at the cellular or protein level, and these are biological causes e.g., following the production of monoamines – neurotransmitters, such as serotonin, noradrenaline or dopamine, disruption of the serotonin and associated enzymes transport, formation of inflammatory processes in the limbic system of the brain; (2) exogenous – activating or being a “self-dependent" causes of depression, e.g., somatic diseases such as hormonal disorders, cancer, surgical procedures, incurable and chronic diseases, deficiencies of B group vitamins, CNS diseases such as multiple sclerosis, Alzheimer’s disease, Parkinson’s disease, Huntington’s chorea, cerebrovascular diseases (especially temporal lobe and frontal lobe), the use of certain drugs and psychoactive substances (Kramer, 2002).

Not all patients suffering from depression respond to the pharmacological treatment. It was demonstrated, that only less than one third of depression patients reach remission after 12 weeks of initial antidepressant treatment (Trivedi et al., 2006), while another 30% of MDD patients are eventually diagnosed with drug treatment resistant disorder (Fitzgerald et al., 2003; Bewernick and Schlaepfer, 2015; Silverstein et al., 2015). In such drug-resistant cases other therapeutic approaches are needed. Numerous studies have shown that repetitive transcranial magnetic stimulation (rTMS) produced significant clinical effects in patients with various neurological and psychiatric disorders, in particularly in depression (Pascual-Leone et al., 1996; Rossi et al., 2009; Rossi, 2013; Lefaucheur et al., 2014). rTMS can be regarded as an adjunctive therapy to the usual pharmacotherapy with the aim of improving or accelerating the efficacy of these treatments by changing brain activity patterns and promoting cortical plasticity (Friston, 1996; Draganski and Kherif, 2013; Dukart et al., 2014). rTMS is usually applied over the dorsolateral prefrontal cortex (DLPFC), which was proven to be clinically effective in resistant depression therapy (Fitzgerald et al., 2003; Avery et al., 2006; O’Reardon et al., 2007; Concerto et al., 2015; Blumberger et al., 2016; Kito et al., 2016; Health Quality Ontario (2016); Filipcic et al., 2017; Teng et al., 2017). The use of two frequencies are recommended to change the local cortical activity: low frequency (LF) rTMS at 1 Hz to reduce neural excitability, and high frequency (HF) rTMS at 10 Hz to enhance neural excitability (Siebner and Rothwell, 2003; Lefaucheur et al., 2014). Considering asymmetry in frontal cortex activity in patients with drug resistant depression, HF rTMS is applied to the left DLPFC, while LF rTMS is targeted to the right DLPFC (Klein et al., 1999; George et al., 2000; Speer et al., 2000). The effectiveness of therapy depends on other factors also. The main experimental factors that introduced variability in reported rTMS effects are the pulse parameters (Arai et al., 2005; Taylor and Loo, 2007; Classen and Stefan, 2008), the different ways of targeting the DLPFC between experimenters and the different anatomy of the underlying gyri between subjects (Thielscher et al., 2011). The rate of responders increases significantly when the number of sessions is greater than 10, the total number of stimuli per session is greater than 1000, and the stimulation intensity is greater than 100% of the resting motor threshold (Gershon et al., 2003; Berlim et al., 2013; Lefaucheur et al., 2014). A minimum of 10 sessions in 1–2 weeks is usually carried out. The duration of the effect was rarely described and no study assessed the long-term effects of rTMS (Lefaucheur et al., 2014).

Considering high temporal resolution of EEG, its low price and easy application, this technique was used very widely to study both the nature of psychiatric disorders and the effect of rTMS. Many investigators have studied the relation between the therapeutic effect of rTMS in depression and spectral power dynamics of various EEG bands (Pozzi et al., 1993; Kwon et al., 1996; Griskova et al., 2007; Spronk et al., 2008; Valiulis et al., 2012; Woźniak-Kwaśniewska et al., 2015), however, these results were not consistent. Moreover, earlier studies demonstrated that EEG complexity analysis using Higuchi’s fractal dimension (FD) can be successfully used in many clinical applications (Klonowski et al., 2000, 2002, 2004, 2005, 2006; Olejarczyk, 2007, 2011; Olejarczyk et al., 2009; Zappasodi et al., 2014, 2015; Cottone et al., 2016, 2017). Some authors applied the FD to compare the complexity of EEG signals in patients with depression and in healthy controls (Bahrami et al., 2005; Ahmadlou et al., 2012; Bachmann et al., 2013, 2018; Akar et al., 2015). All these studies showed a higher FD in both groups of patients with depression (MDD and BP), which would suggest that FD could be a good marker of the rTMS therapy effectiveness. Taking into account the results of these studies, we expected that FD would decrease under the influence of rTMS.

Despite the increasing use of rTMS in drug resistant depression treatment, its exact therapeutic mechanism still remains unknown. The effects of stimulation vary significantly between the studies and individuals. Some drug resistant patients respond well to rTMS treatment, while others remain unaffected. New markers are still needed for more effective patient selection and evaluation of rTMS therapy progress.

The aim of this work was to evaluate the effectiveness of the rTMS applied to the left DLPFC in both groups of patients (MDD and BP). For this purpose, the Higuchi’s Fractal Dimension (FD), were analyzed using high-density EEG signals. The differences between MDD and BP patients as well as between patients responding positively and patients not responding to rTMS therapy in each of these groups separately, were studied. The impact of rTMS was evaluated in individual groups of patients for whole frequency range as well as for every band separately. The topographical differences were also considered. Finally, the dependence of these results on duration of stimulation was studied.

## Materials and Methods

### Subjects

This study reuses the data presented in (Woźniak-Kwaśniewska et al., 2015), which contains additional details on the recording conditions. The EEG data were collected in Psychiatry Department of Grenoble University Hospital, after approval by the local ethical committee (ID RCB: 2011-A00114-37). All participants gave a written informed consent.

Two groups of right-handed patients who met Diagnostic and Statistical Manual of Mental Disorder 4th ed. (DSM-IV) criteria for Major Depressive Episode (American Psychiatric Association, 2000), were examined: 10 patients (6 females, age range 32–69, mean 48.7 ± 12.6) suffering from BP and 8 patients (6 females, age range 44–64, mean 52.1 ± 7.8) suffering from MDD. Each of these groups were also divided into responders and non-responders (**Table 1**).

**Table 1:** Clinical characteristic of the participants.

		Number of patients	Age	Illness duration
MDD	Response	4	53,3 ± 5,8	10.3 ± 6.1
	Non-response	4	51,5 ± 7.5	10,3 ± 5.7
BP	Response	6	49 ± 13	14.8 ± 9.5
	Non-response	4	50 ± 10	24 ± 8.7
	Total	18	48 ± 9.7	15,1 ± 9,6

The inclusion criterion was no response to pharmacological treatment of depression using a minimum of two distinctly different classes of antidepressant medications for actual depressive episode (appropriate doses and duration) occurring at the time of enrolment or earlier. Exclusion criteria were: age under 18 years, drug abuse, current comorbid major mental disorders assessed by clinical examination, neurological illness or convulsive disorders, and previous electroconvulsive therapy. All patients were on a range of medications. For bipolar patients, mood stabilizer medication has been unmodified for at least 2 weeks prior to the entry in the study, and remained unchanged throughout the course of the study. No benzodiazepines were administered 2 weeks before and during rTMS treatment. For MDD patients, pre-treatment with an antidepressant and/or mood stabilizer medication has been unmodified for at least 4 weeks prior to the entry in the study, and remained unchanged throughout the course of the study. Only cyanemazine and hydroxyzine were tolerated during the study.

Demographics characteristics (gender and age) and clinical characteristics (illness and episode duration, depression severity) were evaluated for each patient using Montgomery Asberg Depression Rate Scale (MADRS) (Montgomery and Asberg, 1979), 13-item Beck Depression Inventory (BDI-Short Form) (Collet and Cottraux, 1986; Bouvard et al., 1992, Beck et al., 1996) and Clinical Global Impression (CGI). For bipolar patients, maniac or mixed symptoms were evaluated with Young Mania Rating Scale (YMRS) (Young et al., 1978). All patients were assessed at inclusion, before the first EEG recording and after each 5 rTMS sessions by the same senior psychiatrist (David Szekely). The response to rTMS treatment was defined as at least 50% reduction of the baseline MADRS scores. Patients were qualified as remitters when MADRS score was less than 8. If YMRS was more than 15, at inclusion or during the course of rTMS treatment, patients were excluded from the trial. The absolute changes in MADRS scores between baseline and the end of rTMS (4 weeks after the first evaluation) were used to calculate clinical improvement.

The standard clinical protocol recommended at Grenoble University Hospital was applied. The patients were subjected to rTMS of the left DLPFC over a period of 4 weeks. The rTMS therapy consisted of 20 sessions, with 2000 pulses per session continuously applied at 120% motor threshold. The 64-channel EEG signals were recording immediately before and after the 1st, 10th and 20th session, with FCz as the reference electrode. During the EEG acquisition patients were seated in a reclining armchair with neck and back supported with a pillow, arms relaxed and eyes closed.

### EEG Registration and Preprocessing

Fifteen-minutes resting state with eyes closed pre- and post-rTMS recording, without artifacts were analyzed. EEG data pre-processing was performed using EEGlab and SPM8 tools available in MATLAB software. First EEG data were resampled at 250 Hz and band-pass filtered between 0.5 and 45 Hz. Such prepared data were reviewed for large muscle artifacts and non-stereotypical artifacts. Moreover, the mean of each data channel was removed before the application of an Independent Component Analysis (ICA), which is a method that allows to separate source signals from a multivariate measured signals assuming that the source signals are independent and non-Gaussian. For example, we can use ICA to remove electrooculographic (EOG) or electrocardiographic (ECG) artifacts from EEG signals (Arad et al., 2018). First 10 min of artifacts-free signals were selected to further analysis. These signals were segmented into 20 s successive epochs, which means that each 10 min recording was divided into 30 epochs. The EEG signals were analyzed in whole band as well as in five frequency bands (delta: 1–3 Hz, theta: 4–7 Hz, alpha: 8–12 Hz, beta: 13–30 Hz, gamma: 30–45 Hz) separately.

### Higuchi Fractal Dimension

A fractal dimension (FD) is a measure of signal complexity. The term “fractal” was first introduced in 1975 by Mandelbrot and was described as a set of points that when looked at smaller scales, resembles the whole set. There are many available algorithms to calculate FD, one of them is Higuchi’s fractal dimension (HFD), which is defined in time domain. FD value is always between 1 (for deterministic curves) and 2 (for stochastic signals) (Higuchi, 1988).

The signal is represented by a sequence X(1), X(2), …, X(N), where N is the total number of samples in the epoch. From the given epoch k new sub-epochs *X*_m_^k^ are defined as:

$$\begin{array}{r} X_{\text{m}}^{\text{k}}:X(m),X(m+k),\ldots ,X\left(m+\mathrm{int}\left(\frac{N-m}{k}\right)k\right),\\ m=1,2,\ldots ,k, \end{array}$$

where m – initial time, k – interval time.

For each of the sub-epochs *X*_m_^k^, the average length *L*_m_(k) is computed as:

$$L_{\text{m}}^{\text{k}}=\frac{1}{k}\left[\sum_{\text{i}=1,\text{int}\left(\frac{\text{N}-\text{m}}{\text{k}}\right)}^{\text{k}}|X(m+\mathrm{ik})-X(m+(i-1)k|\frac{N-1}{\mathrm{int}\left(\frac{N-m}{k}\right)}\right]$$

where N is the total number of signal samples, $\frac{N-1}{\mathrm{int}\left(\frac{N-m}{k}\right)}$ is a normalization factor.

The length of the segment L(k) for the time interval k is computed as the mean of the k values, for m = 1, 2,…, k, that is:

$$L(k)=\frac{1}{k}\sum_{\text{m}=1}^{\text{k}}L_{\text{m}}(k)$$

The curve L(k) has a fractal dimension D_f_:

$$L(k)∼k^{-{\text{D}}_{\text{f}}}$$

The calculation is repeated for k values ranging from 1 to *k*_max_. In this work, *k*_max_ was equal 16. The fractal dimension was calculated as the slope of the line being the linear regression coefficient determined by the least squares method.

$$l\eta L(k)∼D_{\text{f}}l\eta \frac{1}{k}$$

### Statistical Analysis

The analysis of variance (ANOVA) with factors: CONDITION (pre- and post-rTMS), GROUP (BP_non-responders, BP_responders, MDD_non-responders, MDD_responders), BAND (delta, theta, alpha, beta, gamma), CHANNEL (1–63), SESSION (session 1st, 10th and 20th) was performed for FD.

In case of significant effects, *post hoc* tests (Tukey HSD) were performed. The statistical threshold was set at *p* < 0.05, with correction for multiple comparisons by controlling the family wise error (FWE).

## Results

The Higuchi fractal dimension was investigated for each of the electrodes in both individual frequency bands (delta, theta, alpha, beta and gamma) as well as in the entire frequency range (0.5–45 Hz).

### Differences Between Conditions After and Before Stimulation

First, the three-way ANOVA with factors CONDITION, BAND and CHANNEL was performed to evaluate the effect of rTMS therapy in four groups of patients (BP_non-responders, BP_responders, MDD_non-responders, MDD_responders).

The differences between conditions, before and after stimulation (factor CONDITION), were found in groups: BP_non-responders, BP_responders and MDD_responders (c.f. **Table 2**).

**Table 2:** The results of ANOVA analysis for factor CONDITION (before and after stimulation) in four groups of patients (BP_non-responders, BP_responders, MDD_non-responders, MDD_responders).

Group	ANOVA results		Mean ± Std	
	F-value	*p*-value	before	after
BP_non-responders	*F*(1,6930) = 75,397	*P* < 0,0001	1.562 ± 0.001	1.579 ± 0.001
BP_responders	*F*(1,9450) = 11,930	*P* = 0.0006	1.543 ± 0.001	1.536 ± 0.001
MDD_non-responders	*F*(1, 5040) = 0.683	*P* = 0.409	1.459 ± 0.001	1.457 ± 0.001
MDD_responders	*F*(1,6930) = 59,239	*P* < 0.00001	1.518 ± 0.002	1.544 ± 0.002

**FIGURE 1:**
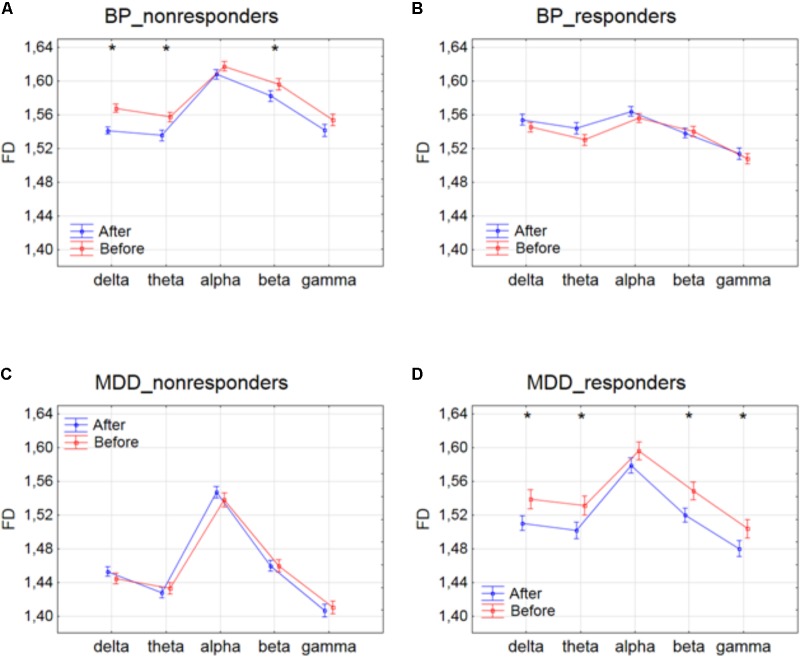
Higuchi fractal dimension for BP_non-responders **(A)**, BP_responders **(B)**, MDD_non-responders **(C)** and MDD_responders **(D)** before and after stimulation in each of frequency bands. The interaction between factors CONDITION and BAND (all three sessions were included). Significant differences between conditions for the individual frequency bands, evaluated by *post hoc* Tukey HSD test, were marked with asterisks.

The differences between conditions, before and after stimulation, for the individual frequency bands: delta, theta, alpha, beta and gamma in each of four groups are shown in **Figure 1**. The interaction between factors CONDITION and BAND was significant only for BP_non-responders group [*F*(4,6930) = 2.627; *p* = 0.033]. Significant differences between conditions for the individual frequency bands, evaluated by *post hoc* Tukey HSD test, were marked with asterisks (c.f. **Figure 1**). For BP_non-responders group the decrease in FD value after stimulation was found mainly in delta, theta and beta bands (**Figure 1A**). No significant FD differences after stimulation were observed in any of the frequency bands in BP_responders and MDD_non-responders (**Figures 1B,C**). For MDD _responders lower values of FD were found after stimulation for each of frequency bands, except alpha band (**Figure 1D**). No significant topographical differences were found between conditions (factors: CONDITION x CHANNEL and CONDITION x BAND x CHANNEL) (c.f. **Supplementary Figure S1**). FD changed similarly in the whole brain. Thus, the factor CHANNEL has not been considered in the further analysis described in subsections 3.2 and 3.3.

### Changes Across Consecutive Sessions

Next, the three-way ANOVA with factors: SESSION, CONDITION and BAND was applied in four groups separately to study the influence of stimulation time on the effect of rTMS therapy. Changes in FD values were analyzed before and after the 1st, 10th and 20th session of rTMS stimulation.

**FIGURE 2:**
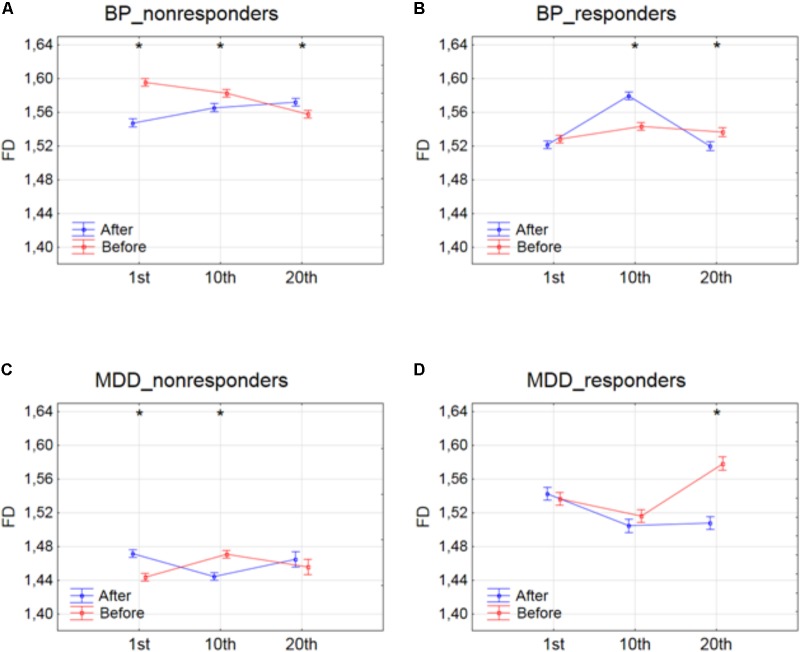
Higuchi fractal dimension for BP_non-responders **(A)**, BP_responders **(B)**, MDD_non-responders **(C)** and MDD_responders **(D)** before and after stimulation in successive sessions (1st, 10th, and 20th session). The interaction between factors CONDITION and SESSION (all frequency bands were included). Significant differences between conditions for the individual sessions, evaluated by *post hoc* Tukey HSD test, were marked with asterisks.

The effect of interaction between factors SESSION and CONDITION was illustrated in **Figure 2**. Significant differences between conditions for every session, evaluated by *post hoc* Tukey HSD test, were marked with asterisks (c.f. **Figure 2**). In BP_non-responders, the FD value significantly decreased after 1st session (**Figure 2A**). Afterward, the FD value started to return to its previous state but slightly decreased again after 10th session. Then, after the 20th session a significant increase of the FD occurred reaching the same level as after the 10th session. In BP_responders, FD value did not change after the 1st session. Afterward, the FD value increased in the second session. However, in the 20th session FD came back to the initial level (**Figure 2B**). In MDD_non-responders, the FD value significantly increased after 1st session (**Figure 2C**) but after 10th session the FD value decreased again reaching the initial level. The FD value did not change after the 20th session. The final FD value after the last session was slightly higher than at the beginning of therapy (**Figure 2C**). The MDD_responders group did not react to the therapy after 1st and 10th session. Nevertheless, after 20th session, the FD value significantly decreased after stimulation (**Figure 2D**).

**FIGURE 3:**
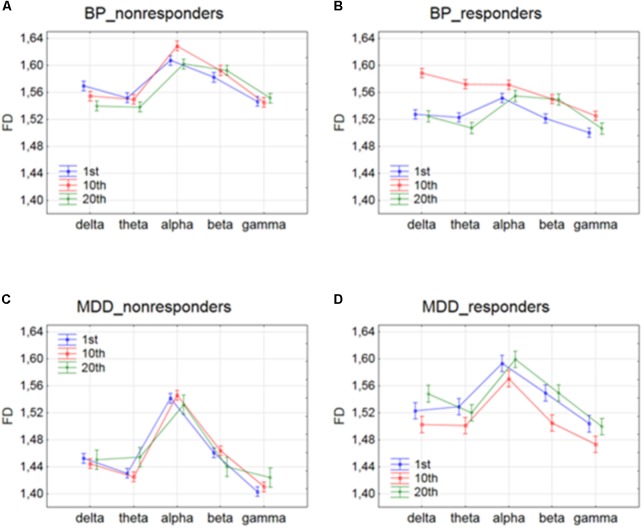
Higuchi fractal dimension for BP_non-responders **(A)**, BP_responders **(B)**, MDD_non-responders **(C)** and MDD_responders **(D)** in five frequency bands. Comparison of results between 1st and 10th, 1st and 20th, and 10th and 20th session. The interaction between factors BAND and SESSION (both conditions were included).

The effect of interaction between factors SESSION and BAND was shown in **Figure 3**. For BP_non-responders group, the FD value decreased for delta band and increased for beta band only between 1st and 20th session (c.f. **Figure 3A**). The FD for alpha band increased after 1st session but then returned to the previous level after 10th session. For MDD_non-responders group, FD values increased for theta band only between 10th and 20th session (c.f. **Figure 3C**). For BP_responders, FD values increased between 1st and 10th session in each of five frequency bands, but then decreased below the initial level in the 20th session in delta, theta, alpha and gamma bands (c. f. **Figure 3B**). For beta band, the value of FD remained unchanged between 10th and 20th session. For MDD_responders, decrease of FD value was found in higher frequency bands (beta and gamma) between 1st and 10th session (c.f. **Figure 3D**). However, between 10th and 20th session FD value increased again in these bands reaching the previous level. For delta band, the value of FD increased between 10th and 20th session.

**FIGURE 4:**
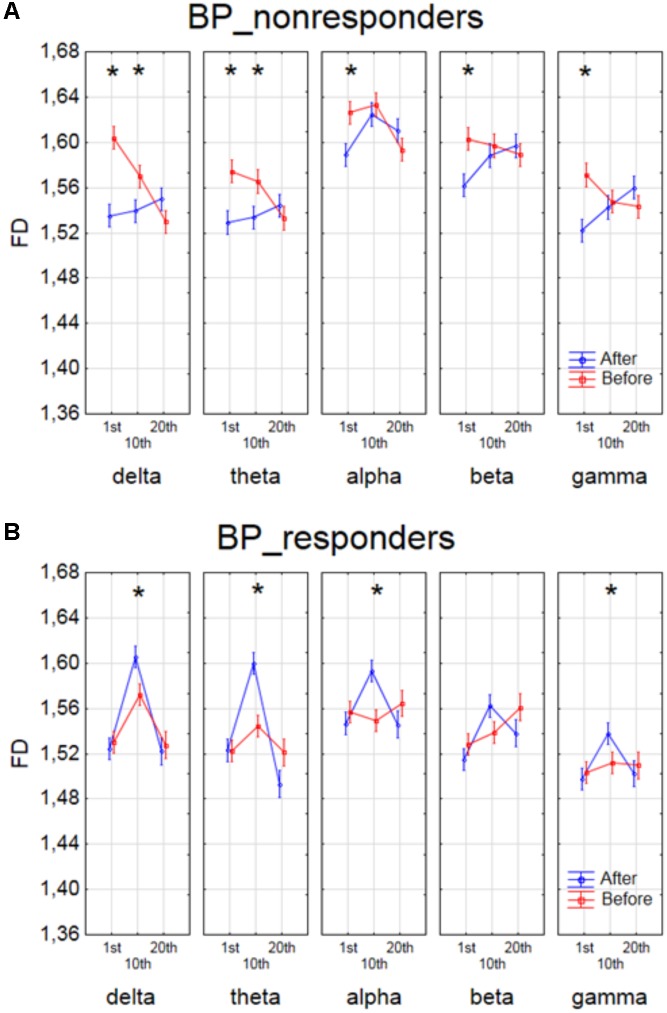
Higuchi fractal dimension for BP_non-responders group **(A)** and for BP_responders group **(B)** for each band separately, showing changes in FD values before and after 1st, 10th and 20th session. The interaction between factors CONDITION, BAND and SESSION (separately for every group of BP patients). Significant differences between conditions for the individual sessions and bands, evaluated by *post hoc* Tukey HSD test, were marked with asterisks.

**FIGURE 5:**
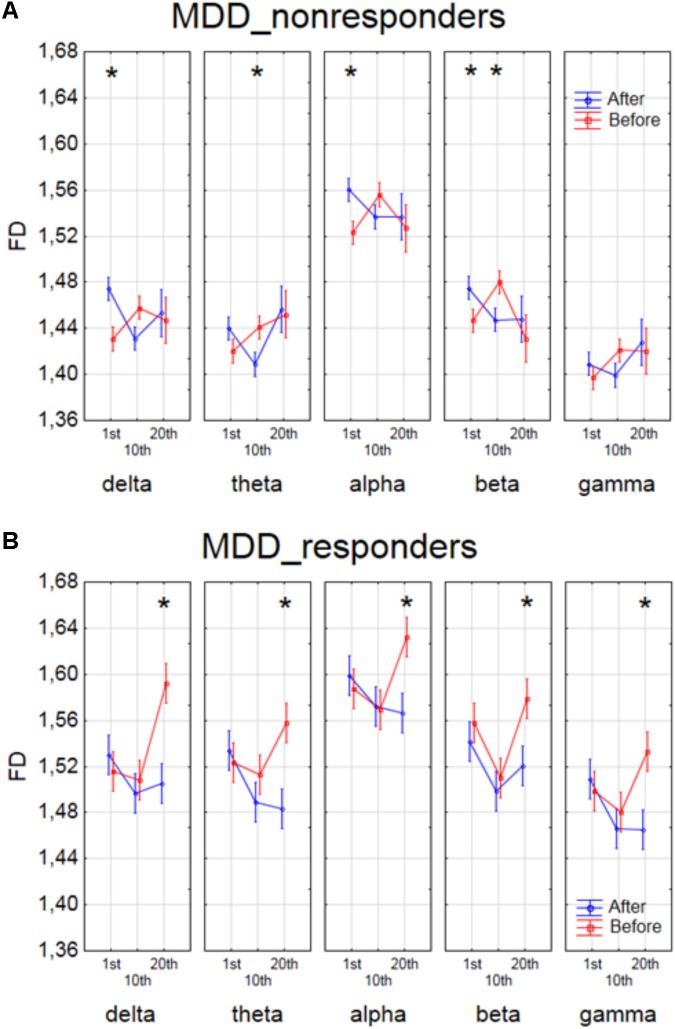
Higuchi fractal dimension for MDD_non-responders group **(A)** and for MDD_responders group **(B)** for each band separately, showing changes in FD values before and after 1st, 10th and 20th session. The interaction between factors CONDITION, BAND and SESSION (separately for every group of MDD patients). Significant differences between conditions for the individual sessions and bands, evaluated by *post hoc* Tukey HSD test, were marked with asterisks.

The effect of interaction between factors SESSION, CONDITION and BAND was shown in four groups of patients in **Figures 4**, **5**. The analysis for BP_non-responders in each of five frequency bands showed a significant decrease of FD value after 1st session and a decrease of low frequency bands (delta and theta) after 10th session (**Figure 4A**). The analysis for BP_responders showed a significant increase of FD value in each of five frequency bands, except beta band, after the 10th session, followed by decrease of FD value after 20th session for all bands, reaching lower level than before 1st session (**Figure 4B**). For MDD_non-responders group the significant increase of the FD values was found after 1st session in delta, alpha and beta bands, and after 10th session in theta and beta bands (**Figure 5A**). For each frequency band, the final FD value did not changed significantly after 20th session in comparison to the FD value before 1st session. For MDD_responders, no significant differences after 1st and 10th sessions were found (**Figure 5B**). The effectiveness of the therapy appeared only after the 20th session. The significant decrease of FD value was noted after the 20th session in each of five frequency bands.

### Differences Between Groups of Patients

**FIGURE 6:**
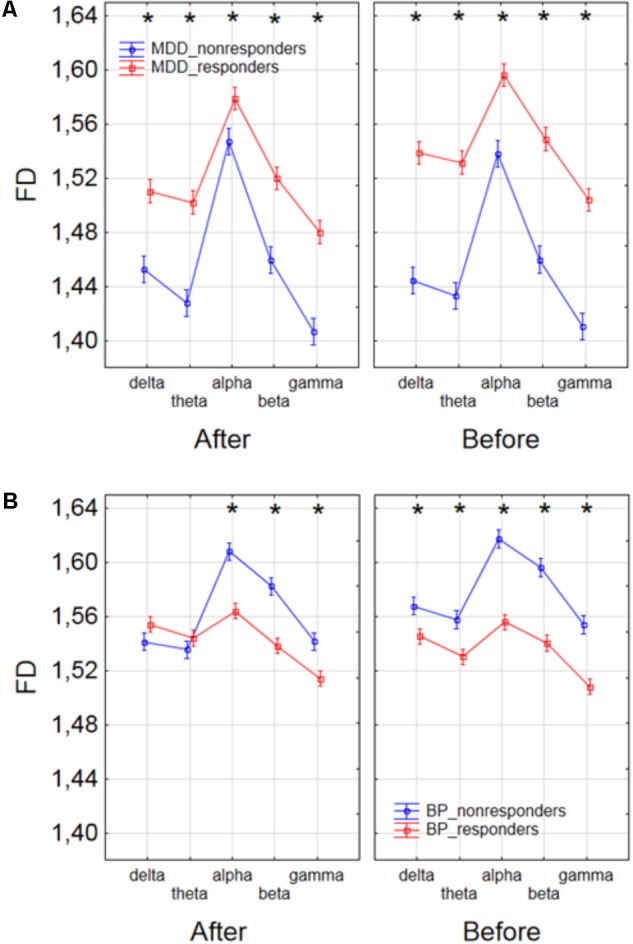
Comparison of FD value between MDD_non-responders and MDD_responders groups **(A)** and between BP_non-responders and BP_responders groups **(B)** in each frequency band separately, for condition before and after session. The interaction between factors CONDITION, BAND, and GROUP (comparison between responders and non-responders separately in groups of MDD and BP patients). Significant differences between both groups for the individual frequency bands and conditions, evaluated by *post hoc* Tukey HSD test, were marked with asterisks.

**FIGURE 7:**
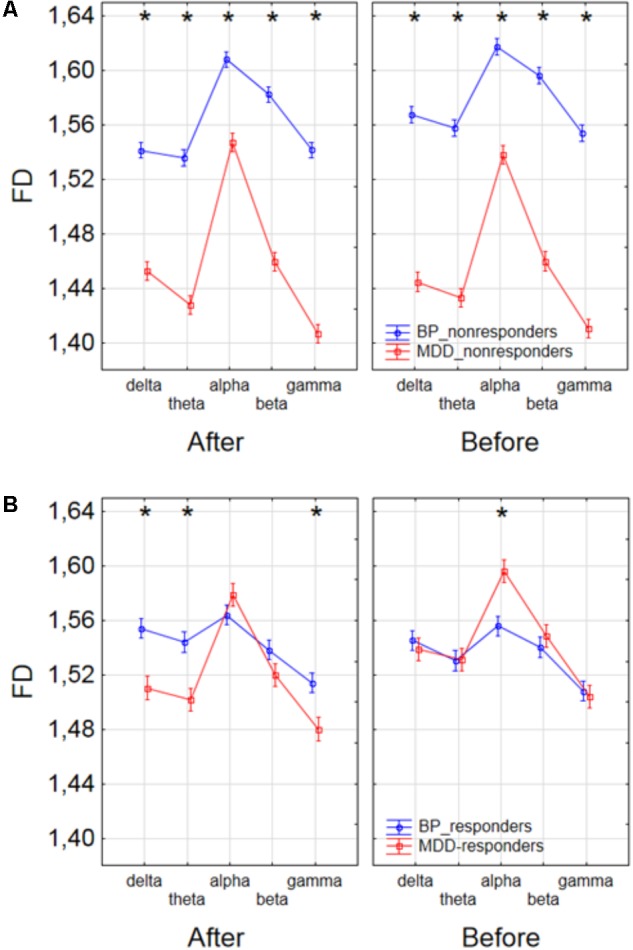
Comparison of FD value between BP_non-responders and MDD_non-responders groups **(A)** and between BP_responders and MDD_responders groups **(B)** in each frequency band separately, for condition before and after session. The interaction between factors CONDITION, BAND and GROUP (comparison between groups of MDD and BP patients separately for responders and non-responders). Significant differences between both groups for the individual frequency bands and conditions, evaluated by *post hoc* Tukey HSD test, were marked with asterisks.

Finally, to find the differences between groups of patients, the three-way ANOVA with factors: GROUP, CONDITION and BAND was performed. The following groups of patients were compared: MDD-responders vs. MDD-non-responders; BP-responders vs. BP-non-responders; MDD-responders vs. BP-responders, and MDD-non-responders vs. BP-non-responders. In MDD_responders higher value of FD was found for each of frequency bands in comparison to MDD_non-responders before as well as after stimulation. For both of groups the highest value of FD was observed for alpha band. The significant decrease of FD value in each frequency band can be observed for MDD_responders group also (compare red lines in right panel with left panel in **Figure 6A**). FD values were lower in BP_responders than in BP_non-responders for higher frequencies (alpha, beta, and gamma bands) after and also before the stimulation (**Figure 6B**). Whereas, for delta and theta band FD was lower only before the stimulation. The significant differences between MDD_non-responders and BP_non-responders were found for each frequency band. The lower FD values were observed in MDD_non-responders for both conditions, after and before the stimulation (**Figure 7A**). The results of the comparison of FD value between BP_responders and MDD_responders were statistically significant for delta, theta and gamma bands after the stimulation. The FD was lower for these bands in group of MDD_responders. Before the stimulation only differences in alpha band were statistically significant. The FD value was higher for MDD_responders. For other frequency bands no significant differences were found between these groups (**Figure 7B**).

## Discussion

The analysis of FD confirmed the effectiveness of rTMS therapy in MDD and BP. Bahrami et al. (2005) and Bachmann et al. (2013, 2018) found that FD in BP patients was higher than FD in healthy controls. Thus, we expected that FD would decrease in BP after stimulation. The patients from group of BP_responders reacted negatively to the therapy in the 10th session, but the change was not permanent (**Figure 4B**). Changes of FD showed an unfavorable reaction in the initial phase of therapy, but finally, the therapy was effective because the FD values after the 20th session were lower for each of five frequency bands than before the 1st session (c. f. **Figure 4B**), what was expected. This may mean that patients suffering from BP need longer therapy to be effective the stimulation. However, further studies are required to confirm these results.

In MDD_responders, FD decreased in each of frequency bands after 20th session (c.f. **Figure 5B**). This result is in line with the conclusions of other authors (Ahmadlou et al., 2012; Bachmann et al., 2013, 2018; Akar et al., 2015). The authors found the higher FD values in the MDD group in beta and gamma bands in the frontal lobe, than in the control group, that means the decrease of FD after stimulation is desirable in this group of patients.

In MDD_non-responders, the response to rTMS was opposite to the expected one at the beginning of the therapy. However, the final FD value did not changed significantly in comparison to the initial FD value.

In BP_non-responders the decrease in FD value after 1st session was found in each of five frequency bands as well as a decrease of low frequency bands (delta and theta) after 10th session (**Figure 4A**). No significant changes were observed in the last session. Thus, it can mean that the patients from the BP_non-responders group reacted positively to a shorter rTMS therapy.

Our results of the analysis of Higuchi fractal dimensions showed that rTMS stimulation can be an effective therapy in patients with MDD and bipolar disorder, however, some differences in response to the therapy across individual groups were found.

## Conclusion

In this paper, the impact of rTMS on the complexity of EEG evaluated by FD was studied for the first time. We demonstrated that the complexity analysis of EEG data in persons with depression subjected to rTMS allowed to find the differences between conditions (before and after stimulation) and between individual groups of patients (MDD and BP, responders and non-responders) as well as to evaluate the impact of time stimulation on these results. The results of other authors (Bahrami et al., 2005; Ahmadlou et al., 2012; Bachmann et al., 2013, 2018; Akar et al., 2015) showed that FD is higher in both groups of patients with depression (MDD and BP) than in healthy controls. Thus, FD can be a good marker of rTMS efficiency because its changes allow to unambiguously conclude whether the effect of stimulation is positive or negative as well as allow to evaluate an optimal time of rTMS. Nevertheless, this study has some limitations. A bigger number of patients in each group as well as group of healthy controls should be examined in the future studies to confirm these preliminary but promising results.

Moreover, the FD is only one of many measures of the complexity of EEG signal. The complexity can be infer by studying of interactions between signals. The interactions between the EEG signals can be evaluated by different connectivity measures and indices based on graph theory (Olejarczyk et al., 2017a,b; Olejarczyk and Jernajczyk, 2017). Recently, a new field of Network Physiology has been developed (Bartsch et al., 2015; Liu et al., 2015; Ivanov et al., 2016). Its objective is an investigation of interactions not only within the brain but also between the brain and other organs by the analysis of signals from different non-linear dynamic systems in the human organism. This field indicates new directions for future research.

## Ethics statement

This study was carried out in accordance with the recommendations of Ethics Committee of the Grenoble University Hospital with written informed consent from all subjects.

## Data Availability Statement

The datasets for this manuscript are not publicly available because of Grenoble University Hospital policy. Requests to access the datasets should be directed to Prof. Olivier David, email: Olivier.David@inserm.fr.

## Author Contributions

KL analyzed the EEG data and wrote the manuscript. UZ preprocessed the EEG data. EO conceived the work, supervised the EEG data analysis, and wrote the manuscript. AW-K acquired the EEG data. DS recruited the patients and designed the trial. OD designed the trial, interpreted the EEG data, and critically reviewed the manuscript.

## Conflict of Interest Statement

The authors declare that the research was conducted in the absence of any commercial or financial relationships that could be construed as a potential conflict of interest.
