# Supplementary material for: Complexity Analysis of EEG Data in Persons With Depression Subjected to Transcranial Magnetic Stimulation
Source: Front Physiol. 2018 Sep 28;9:1385. doi: 10.3389/fphys.2018.01385 (PMC6172427; doi:10.3389/fphys.2018.01385)
Supplement: Supplementary file 1 [file Data_Sheet_1.PDF]

Figure S1. Higuchi fractal dimension for BP_nonresponders (A), BP_responders (B), MDD_nonresponders (C) and MDD_responders (D) before and after stimulation for every EEG channel. The interaction between factors CONDITION and CHANNEL (five frequency bands and three sessions were included).

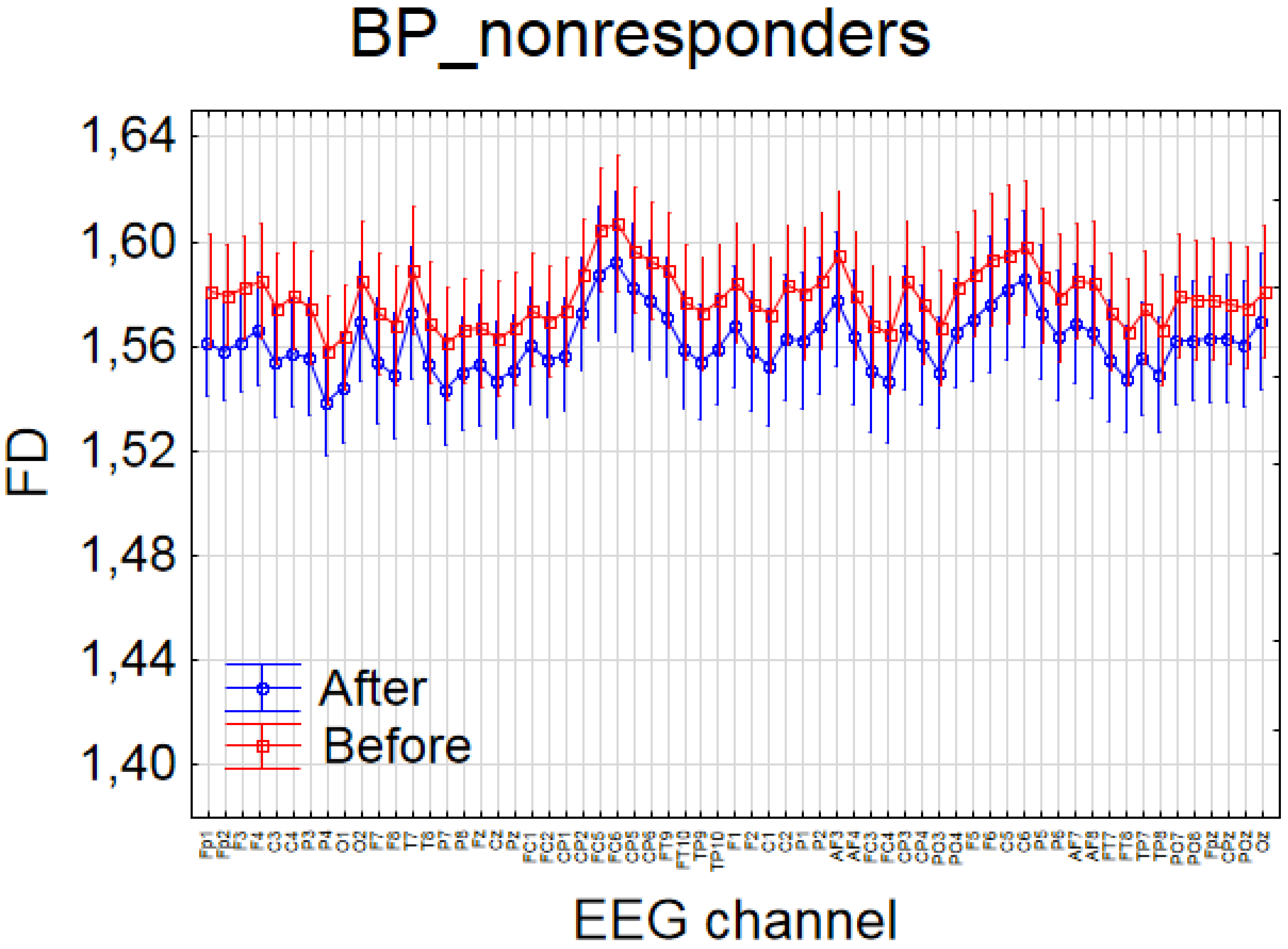
BP_nonresponders
FD
1,64
1,60
1,56
1,52
1,48
1,44
1,40
EEG channel
After
Before

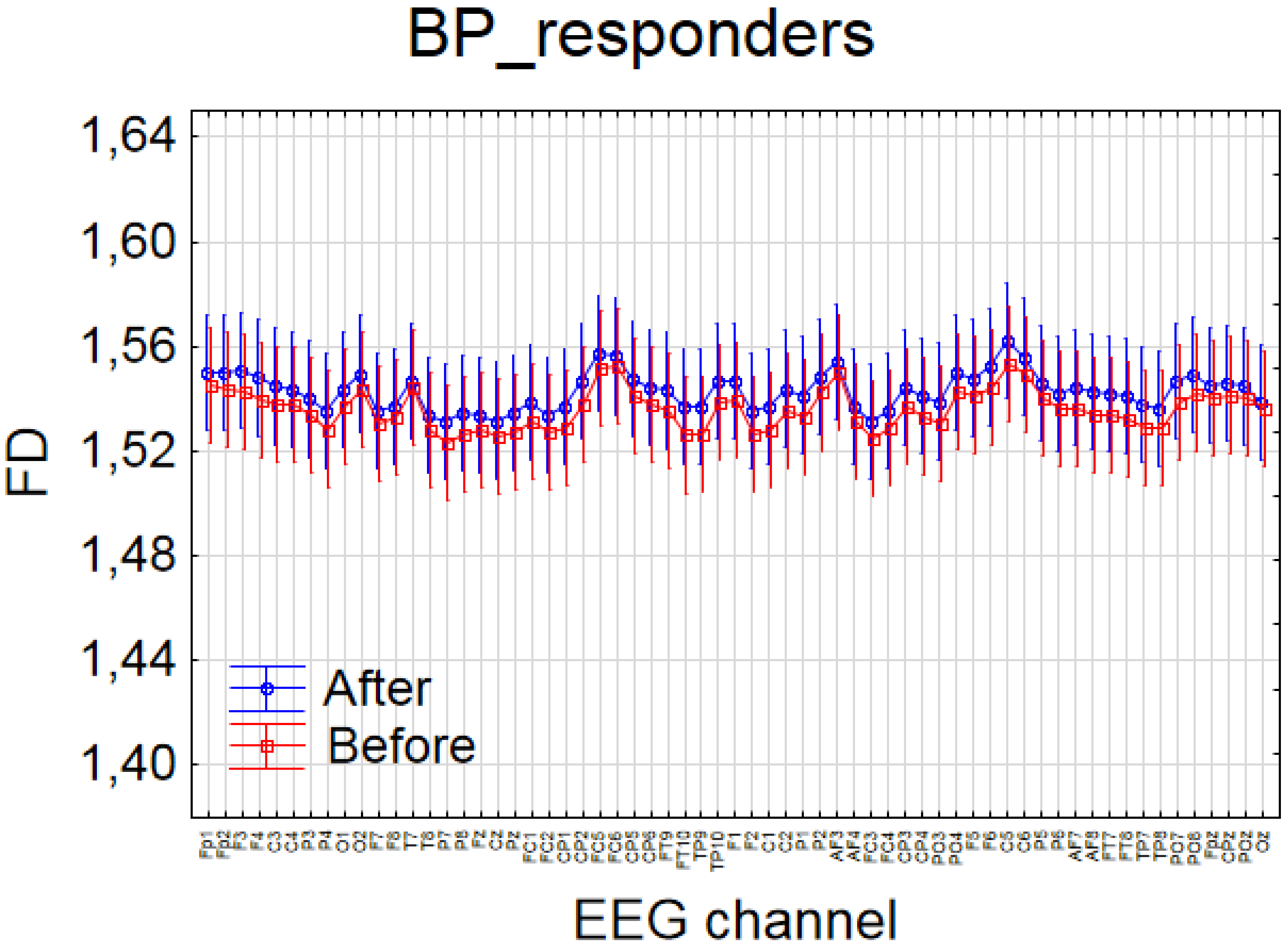
BP_responders
1,64
1,60
1,56
1,52
1,48
1,44
1,40
FD
After
Before
Fp1 Fp2 F3 F4 C3 C4 P3 P4 O1 O2 F7 F8 T7 T8 P7 P8 Fz Cz Pz FC1 FC2 CP1 CP2 FC5 FC6 CP5 CP6 FT9 FT10 TP9 TP10 F1 F2 C1 C2 P1 P2 AF3 AF4 FC3 FC4 CP3 CP4 PO3 PO4 F5 F6 C5 C6 P5 P6 AF7 AF8 FT7 FT8 TP7 TP8 PO7 PO8 Fpz CPz POz Oz
EEG channel

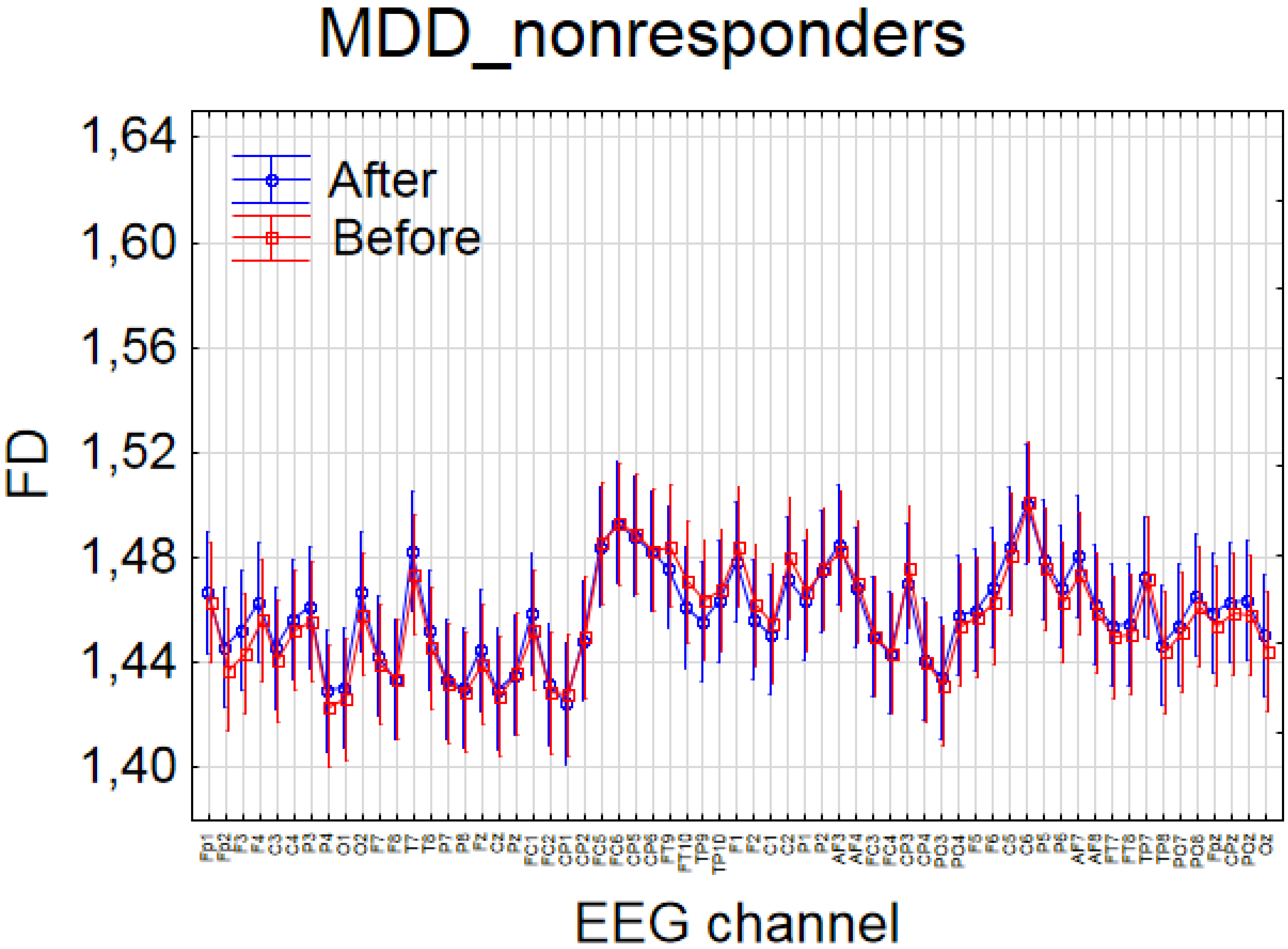
MDD_nonresponders
After
Before
FD
1,64
1,60
1,56
1,52
1,48
1,44
1,40
EEG channel

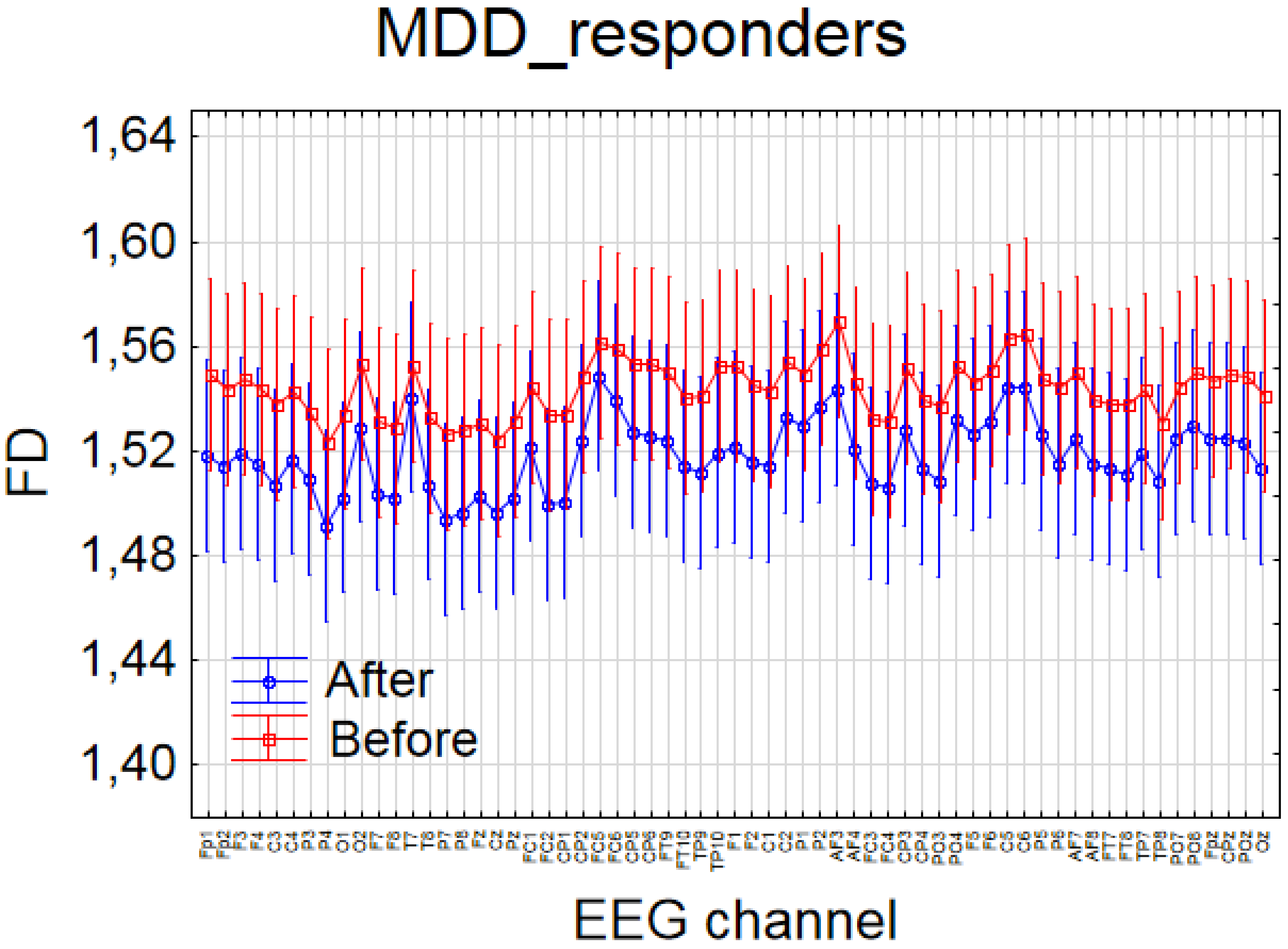

MDD_responders
FD
1,64
1,60
1,56
1,52
1,48
1,44
1,40
After
Before
Fp1 Fp2 F3 F4 C3 C4 P3 P4 O1 O2 F7 F8 T7 T8 P7 P8 Fz Cz Pz FC1 FC2 CP1 CP2 FC5 FC6 CP5 CP6 FT9 FT10 TP9 TP10 F1 F2 C1 C2 P1 P2 AF3 AF4 FC3 FC4 CP3 CP4 PO3 PO4 F5 F6 C5 C6 P5 P6 AF7 AF8 FT7 FT8 TP7 TP8 PO7 PO8 Fpz CPz POz Oz
EEG channel
